# Assessment of Double Outlet Left Ventricle in Pediatrics Using Transthoracic Echocardiography and Computed Tomographic Angiography

**DOI:** 10.1002/pdi3.70005

**Published:** 2025-05-08

**Authors:** Xu Zhu, Guangyi Zhong, Xiaojuan Ji, Xue Xiang, Min Zheng, Huina Yan, Rui Li

**Affiliations:** ^1^ Department of Ultrasound The Third Affiliated Hospital of Chongqing Medical University Chongqing China; ^2^ Department of Ultrasound Children's Hospital of Chongqing Medical University Chongqing China; ^3^ Ministry of Education Key Laboratory of Child Development and Disorders Chongqing Key Laboratory of Child Infection and Immunity International Science and Technology Cooperation Base of Child Development and Critical Disorders Chongqing China; ^4^ Department of Pediatrics Yilong County General Hospital Sichuan China; ^5^ Department of Ultrasound Chongqing General Hospital Chongqing China

**Keywords:** clinical diagnosis, computed tomographic angiography, double outlet left ventricle, pediatrics, transthoracic echocardiography

## Abstract

Double outlet left ventricle (DOLV) is a rare congenital cardiac anomaly in which both great arteries originate entirely or predominantly from the morphologic left ventricle. The aim of this study is to explore its clinical presentations and compare the diagnostic accuracy of transthoracic echocardiography (TTE) with computed tomographic angiography (CTA) on DOLV. We took TTE as a first‐line examination modality, considering CTA and surgical results as the gold standard. Ten suspected patients with DOLV were identified at the Children's Hospital of Chongqing Medical University from March 2009 to November 2023. The clinical presentations, TTE, CTA, and follow‐up data of the children were analyzed retrospectively. All 10 cases (100%) underwent TTE examination, and 7 patients (70%) received CTA examination. Six patients (60%) ultimately underwent surgery and were confirmed to have DOLV. DOLV were initially diagnosed using TTE with a diagnostic accuracy rate of 85.71% after comparisons with CTA and surgery results. Ventricular septal defect (100%) and atrial septal defect (80%) were the common associated abnormality. The clinical manifestation of DOLV was atypical, and TTE has a relatively high diagnostic accuracy for DOLV in pediatrics, making it a very valuable tool for early detection.

## Graphical abstract

The clinical manifestation of DOLV was atypical. TTE has a relatively high diagnostic accuracy for DOLV in pediatric, which is very valuable for its early detection.

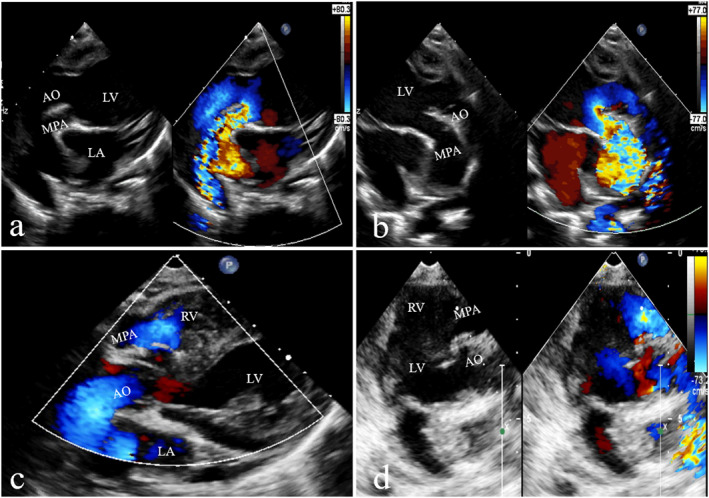

## 1 Introduction

Double outlet ventricles with concordant atrioventricular connections are about 1%–3% in all cases of congenital heart disease [1, 2]. Double outlet left ventricle (DOLV) accounts for less than 5% of double outlet ventricles and is a rare congenital cardiac malformation with a prevalence rate of approximate 1 per 200,000 live births [1, 3, 4]. The first diagnostic anatomy of DOLV was established by Paul in 1970 [5], which is both great arteries arising entirely or predominantly from the left ventricle.

DOLV is quite a complex malformation and its morphology and hemodynamics are extremely variable. At present, it can be diagnosed using transthoracic echocardiography (TTE), computed tomographic angiography (CTA), and digital subtraction angiography (DSA). TTE remains the preferred imaging examination method for diagnosing DOLV with its advantages of noninvasiveness, good repeatability, low cost, and the ability to show vascular malformations [2, 6]. It can also provide detailed information about cardiac remodeling and ventricular function, following surgical repair or catheter intervention, and contribute significantly to clinical management during long‐term follow‐up in pediatrics. DOLV researches have mostly been published in individual case reports because of rarity.

In the study, 10 suspected DOLV cases at Children's Hospital of Chongqing Medical University from March 2009 to November 2023 were retrospectively analyzed, including the data on clinical manifestations, imaging features, treatment, and follow‐up. The aim is to explore the clinical manifestations, evaluate the diagnostic effectivity of TTE, and compare the diagnosis effectivity with CTA.

## 2 Materials and Methods

This retrospective study was approved by the research ethics committee at Children's Hospital of Chongqing Medical University (2023, 511). Informed consent was waived due to the retrospective design.

### 2.1 Patients

A total of 10 children with suspected DOLV were included. The incidence of each type (the ratio of DOLVs of each type vs. the total number of patients) was calculated. Data were collected from medical records, including physical manifestations, TTE, CTA, and surgery reports.

### 2.2 TTE Examination

Patients were examined with TTE using a Philips Q7c, Philips IE33, GE Vivid i color Doppler ultrasonic instrument with a 1–8 MHz transducer. Children less than 3 years old were orally or rectally given chloral hydrate sedative at a dose of 0.5 mL/kg. The patients underwent detailed echocardiography according to Van Praagh segmented diagnosis [5], which was used to observe the heart position, atrioventricular junction, and ventricular large vascular connection, and routinely analyze left heart function.

### 2.3 CTA Examination

Before the CT examination, informed consents for the potential adverse reactions to contrast medium and radiation exposure were obtained from patient guardians. The images were acquired with Lightspeed VCT 64‐slice spiral CT (GE Healthcare, USA) and Brilliance ICT 256‐slice spiral CT (Philips, Netherlands) by using the following parameters: tube voltage, 90–120 kV; automatic tube current, 60–100 mA; pitch, 0.984; slice thickness, 5 mm; and slice interval, 5 mm. Patients were scanned in the supine position from the neck to the diaphragm. A contrast agent (Omnipaque, GE Healthcare, USA) was injected during the scan into the scalp vein or elbow vein of the patient at 300–350 mg/mL at 2–5 mL/s. The CT scanner incorporates postprocessing methods such as multiplanar reconstruction and three‐dimensional volume rendering. All images were reconstructed with a 1.25 mm slice thickness. Multidirectional and multiangle reconstruction images were used to evaluate the structure of the heart and the origin of the blood vessels.

### 2.4 Statistical Analysis

SPSS 25.0 software was used for data analysis. Quantitative data with normal distributions were expressed as mean ± standard deviation (SD). Count data are expressed in terms of examples and percentages. Utilize a descriptive statistical method for analysis.

## 3 Results

In all 10 cases, there were 8 males (80%) and 2 females (20%). The median age was 0.76 year old with an interquartile range of 0.28–2.46 years old. The youngest patient was 14 days old, whereas the oldest patient was 12 years old. The average birth weight was 8.32 ± 4.48 kg. Seven cases were born at full term. Among them, 6 patients (60%) were first admitted to the respiratory department due to symptoms such as coughing, wheezing, cyanosis, or poor activity. During auscultation, a 3/6 level systolic murmur was heard in the 2–4 intercostal space along the left edge of the sternum in 9 cases. The general clinical performance results are summarized in Table 1.

**TABLE 1:** General clinical performance of the double outlet left ventricular (*n* = 10).

Case	Clinical symptoms	Breathing (times/min)	Heart rate (beats/min)	BP (mmHg)	SPO_2_ (%)	ECG	Outcomes
1	Heart murmur	43	155	89/69	/	Right atrial enlargement and left‐axis deviation	Surgery (good recovery)
2	Cyanosis	32	126	95/68	85	Sinus rhythm	Surgery (in good condition)
3	Cyanosis	24	92	85/55	56	Sinus arrhythmia, right atrial enlargement, right ventricular hypertrophy, and first‐degree atrioventricular block	Surgery (in good condition)
4	Heart murmur	32	122	72/48	90	T‐wave changes	Surgery (good recovery)
5	Cyanosis	48	128	85/61	81	Low‐voltage QRS complexes and T‐wave changes	Surgery (died)
6	Heart murmur	24	107	96/60	75	Sinus arrhythmia, right deviation of electrical axis, and T‐wave changes	Surgery (good recovery)
7	Short of breath	60	142	106/20	/	/	Lost to follow‐up
8	Short of breath	70	168	/	78	Sinus arrhythmia, right ventricular hypertrophy, left‐axis deviation, and T‐wave changes	Surgery (good recovery)
9	Constipation, vomiting	35	150	/	/	Sinus arrhythmia and T‐wave changes	Died
10	Cyanosis	54	136	90/35	/	/	Lost to follow‐up

Abbreviations: BP, blood pressure; ECG, electrocardiogram; SPO_2_, peripheral capillary oxygen saturation.

All patients (100%) received TTE examination, seven patients (70%) received CTA examination, and three patients (30%) received DSA examination (Table 2). Six cases of DOLV were first diagnosed using TTE, with a diagnostic accuracy rate of 85.71%, compared with CTA and/or surgery results. One case (10%) was misdiagnosed as the double outlet of right ventricular (DORV).

**TABLE 2:** Echocardiology imaging results of 10 cases of DOLV.

Case	Anomalies	VSD location	VSD size (mm)	Great arteries relationship	Operation	Confirmation
1	ASD/PDA/VSD/ARVD/MGA/TA/PS	Sub‐AO	10	AORA, AO overriding	Glenn + PDA + Fontan	CTA + surgery
2	Dextroversion/ASD/VSD/MGA	Sub‐PA	25	AOL	DOLV correction (internal diploic) + ASD	CTA + DSA + surgery
3	VSD/PFO/MGA/MAPCA/PS	Sub‐PA	18	AOA and PA overriding	Glenn	CTA + DSA + surgery
4	VSD/PDA/MGA/CSSD/PS	Doubly committed	15	AOA and AO overriding	DOLV correction + VSD + PS + Glenn + ASD	CTA + surgery
5	Dextroversion/ASD/VSD/PDA/MGA/MAPCA/PS	Doubly committed	15	AOA	DOLV correction + PDA	CTA + surgery
6	Dextroversion/ASD/VSD/PS/MAPCA/MGA	Sub‐AO	20	AOL	DOLV correction + RV Rastelli + VSD (inner tunnel) and atrial switch (mild mustard) + Glenn + ASD (diploic)	CTA + DSA + surgery
7	ASD/VSD/PDA/CoA/PLSVC	Sub‐PA	9	AORP and PA overriding	/	CTA
8	Extrathoracic/ASD/VSD/LAS	Remote	13.6	AOR	Not quite clear (external hospital)	Surgery
9	Dextrocardia/ASD/VSD/PDA/RAA	Sub‐AO	12.8	AOL	/	Only echo
10	Mesocardiac/ASD/VSD/PDA/ARVD/IAA/PLSVC	Sub‐PA	11	AOP	/	Only echo

Abbreviations: AO, aorta; AOA, aorta directly anterior (to the pulmonary trunk); AOL, aorta to the left (a side‐by‐side positioning to the left of the pulmonary trunk); AOP, aorta directly posterior (to the pulmonary trunk); AOR, aorta to the right (a side‐by‐side alignment to the right of the pulmonary trunk); AORA, aorta to the right anterior (to the pulmonary trunk); AORP, aorta to the right posterior (to the pulmonary trunk); ARVD, arrhythmogenic right ventricular dysplasia; ASD, atrial septal defect; CoA, coarctation of aorta; CSSD, coronary sinus septal defect; CTA, computed tomographic angiography; DSA, digital subtraction angiography, IAA, interruption of aortic arch; LAS, left arterial septum; MAPCA, main aortic and pulmonary collateral vessels; MGA, malposition of the great arteries; PA, pulmonary artery; PDA, patent ductus arteriosus; PFO, patent foramen ovale; PLSVC, persistent left superior vena cava; PS, pulmonary artery stenosis; RAA, right aortic arch; RV, right ventricular; TA, tricuspid atresia; VSD, ventricular septal defect.

### 3.1 TTE

**FIGURE 1:**
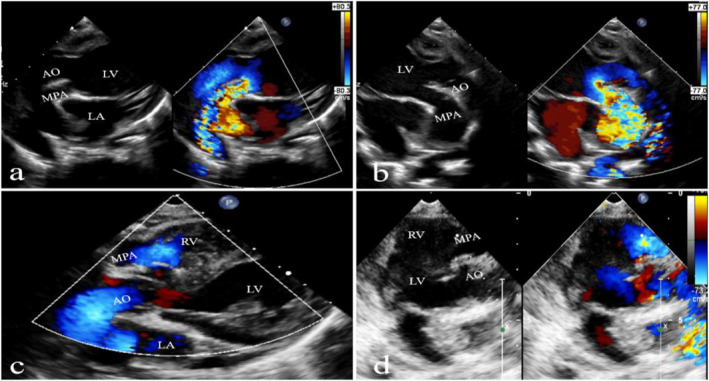
Echocardiographic images in a case of levocardia with the right ventricular loop are presented. Preoperative nonstandard parasternal five chamber view (a) and nonstandard parasternal left ventricular long axis view (b) show that aortic widening straddles the interventricular septum, with the aorta mostly originating from the left ventricle and pulmonary artery stenosis, also arising from the left ventricle. Postoperative images (c and d) show that the aorta connects to the left ventricle and the pulmonary artery connects to the right ventricle, which correspond to the preoperative views in panels (a) and (b), respectively. AO, aorta; LA, left atrium; LV, left ventricle; MPA, main pulmonary artery; RA, right atrium; and RV, right ventricle.

**FIGURE 2:**
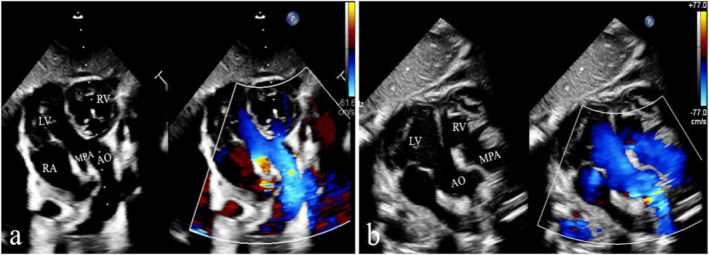
Images of echocardiography with dextroversion and left ventricular loop of case 6. (a) The left ventricular outflow tract view under the xiphoid process shows both the aorta and pulmonary artery originate from the left ventricle on the right. (b) The corresponding postoperative view shows that the aorta originates from the left ventricle on the right and the pulmonary artery connects to the right ventricle on the left. AO, aorta; LA, left atrium; LV, left ventricle; MPA, main pulmonary artery; RA, right atrium; and RV, right ventricle.

The two‐dimensional images captured from TTE showed both great arteries originate entirely or predominantly from the left ventricle (Figures 1 and 2). The common associated abnormalities were ventricular septal defect (100%), atrial septal defect (80%), patent ductus arteriosus (60%), and pulmonary stenosis (50%). As for the cardiac position, approximately 40% of those cases showed the heart located on the left side of the thoracic cavity and 30% of those cases were diagnosed with dextrocardia (a condition where the heart is rotated to the right side of the chest). One patient (10%) had a right aortic arch (Table 2). Ten of the patients in this series did not have a coronary abnormal pattern.

Among these 10 cases, all were complicated by VSD, with 4 cases located in the subpulmonic area (40%), 3 cases in the subaortic area (30%), 2 patients with doubly committed VSD (20%), and 1 patient with a noncommitted or remote VSD (10%).

### 3.2 CTA

**FIGURE 3:**
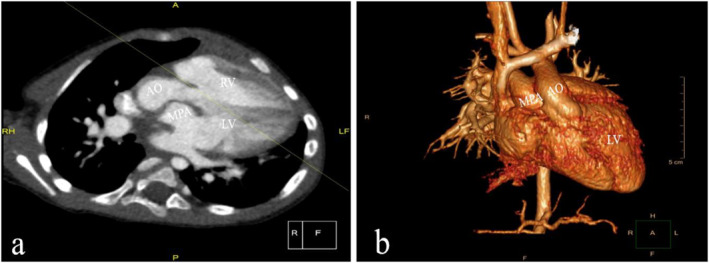
The mediastinal window and the three‐dimensional reconstruction of case 5. (a) The mediastinal window and (b) the three‐dimensional reconstruction show both the aorta and pulmonary artery originate from the left ventricle, and the position of the major artery is abnormal, overriding aorta. AO, aorta; LA, left atrium; LV, left ventricle; MPA, main pulmonary artery; RA, right atrium; and RV, right ventricle.

A three‐dimensional reconstruction from multiple two‐dimensional CTA acquisitions provided the opportunity to evaluate the origin and location of two great arteries (Figure 3) and associated cardiac malformations. CTA was superior to TTE in detecting the spatial structure of large arteries with high spatial and contrast resolution before surgery. CTA can demonstrate the relationship of the great arteries, the location of the VSD, and the size and arborization of the pulmonary arteries [2]. Given the requirement of ionizing radiation exposure, CT is generally reserved for cases that a significant degree of uncertainty persists following TTE in pediatrics.

### 3.3 Surgery Data

Seven out of the ten patients (70%) underwent surgical treatment and DOLV was confirmed during the operation. Six of these surgical cases were operated in our hospital, whereas one patient received surgical treatment at an external hospital. Among the six surgical patients, four underwent biventricular repair of DOLV, one underwent the Glenn procedure alone, and the remaining patient underwent the Fontan procedure following a bidirectional Glenn shunt surgery. Case 2 had minimal atrial‐level shunt after the operation. Case 5 underwent primary surgery but died due to insufficient oxygen saturation while waiting for secondary radical surgery at a local hospital. Two patients were lost to follow‐up after hospital discharge. Patients were mainly followed up using TTE after surgery, with the longest follow‐up time being nearly 14 years.

However, there were no significant differences in left ventricular function in the 6 cases in TTE evaluation before and after surgery.

## 4 Discussion

A double outlet heart is a congenital heart defect characterized by abnormal connections of the ventricular arteries. It is classified into three types: DORV, DOLV, and double outlet both ventricles (DOBV) [2]. Among these, DOLV is particularly rare, featuring a ventriculoarterial connection where both great arteries—the aorta and the pulmonary artery—arise predominantly or entirely from the left ventricle [7]. The embryonic pathogenesis of DOLV can be explained as excessive leftward displacement of the embryonic cone, abnormal growth of the cone, and absorption or misorientation of the inferior part of the interventricular septal artery above the supraventricular crest, separating the right ventricular funnel from the two major arteries [8].

The pathology and associated cardiac defects of patients with DOLV are highly variable [2]. There are two methods for DOLV classification. One method, based on the position of the VSD, is relative to the great arteries in hearts with two ventricles, and it is classified into four types: subaortic VSD, subpulmonic VSD, doubly committed VSD, and remote VSD [9, 10, 11]. The other method, based on the hypoplasia of the right ventricle, is divided into two types: the right ventricular (RV) is normal and therefore suitable for a biventricular repair and the RV is hypoplastic and requires a univentricular repair [6]. In our study, there were 10 DOLV cases, with more males than females. All 10 cases were complicated with VSD. Among them, 4 cases occurred in subpulmonic, three cases were subaortic, two patients presented with doubly committed VSD, and one patient exhibited a noncommitted VSD. Two patients in the second method for DOLV classification were type 2.

DOLV was reported with or without intact ventricular septum, either situs solitus or inversus, concordant, or discordant atrioventricular connections [5, 12, 13]. The position of the aorta in relation to the pulmonary trunk can be divided into six types: a normal positioning to the right posterior (AORP), a side‐by‐side alignment to the right (AOR), a location to the right anterior (AORA), a side‐by‐side positioning to the left (AOL), a positioning directly anterior (AOA), and a positioning directly posterior (AOP). There were five cases that had pulmonary outflow tract obstructions. In our study, there were three cases with discordant atrioventricular connections (Figure 2): atrial orthoposition, ventricular inversion, and two large blood vessels originating from the right ventricle (dissected left ventricle). The more common associated abnormalities were ventricular septal defect (100%), atrial septal defect (80%), patent ductus arteriosus (60%), and pulmonary stenosis (50%).

The clinical presentation of DOLV is based on the underlying anatomy and resulting hemodynamics and depends on the amount of pulmonary blood flow and the location of the outflow tract obstruction. Most patients with DOLV will have pulmonary outflow obstruction, and they present with shortness of breath even cyanosis. When combined with other intracardiac malformations, it will present as cardiac murmurs. Among the 10 cases described, 4 cases presented with cyanosis, 3 cases with heart murmur, 2 cases presented with shortness of breath, and 1 case presented with gastrointestinal symptoms.

It is very important to make comprehensive imaging assessments by using a variety of modalities. Traditionally, angiography was considered indispensable for diagnosing DOLV. Today, TTE provides diagnosis and comprehensive anatomic characterization of this rare anomaly with its advantages of noninvasiveness, good reproducibility, and high compliance [13]. TTE can show the positional relationship with the two great arteries by parasternal long‐axis and subcostal views, the degree of override of the arteries, and the position and size of the VSD and associated cardiac malformations [5, 6]. DOLV was found to be associated with a wide range of cardiac anatomies and hemodynamic findings including excessive pulmonary flow, pulmonary hypoperfusion, and right ventricular dysfunction [6]. TTE can also provide detailed information about cardiac remodeling and ventricular function following surgical repair and contribute significantly to clinical management during follow‐up [14, 15]. CTA is more accurate than TTE in displaying the origin and spatial structure of large arteries with high spatial resolution. However, it is less helpful for the intravascular blood flow assessment and ionizing radiation exposure, when sedated in infants. Imaging assessments often lead to diagnostic confusion, particularly in regard to differentiating between transposition of great arteries (TGA) and DORV, owing to the intricate and varied relationship existing between the great arterial trunk and the VSD. Within our study, 7 cases underwent CTA and the accuracy of TEE for DOLV diagnosis was 85.71%. One case misdiagnosed as DORV for the first time.

Complete repair of DOLV in infancy has been shown to be a safe and feasible surgical option [16]. Timely correction of DOLV can help to normalize the heart's structure and function, potentially reducing the risk of complications in future life. The surgical strategy for DOLV includes biventricular correction, Rastelli repair, pulmonary root translocation, and VSD closure [17, 18, 19]. Biventricular repair is the preferred surgical management option, and univentricular repair is required in the presence of tricuspid atresia or hypoplastic right ventricle [17, 20, 21]. The relative position of the great arteries is indeed a crucial determinant in selecting the appropriate surgical approach for correcting. In the six surgical cases in our hospital, four patients underwent DOLV correction to rectify the ventricular–arterial connection (biventricular repair). Conversely, the remaining two patients received palliative surgeries, such as the Glenn procedure, necessitated by a case of right ventricular hypoplasia and a severe case of pulmonary hypoxemia coupled with hypoxia symptoms. Among the four cases with biventricular repair, two children underwent Glenn's procedure simultaneously due to insufficient perfusion in the pulmonary artery. During the postoperative follow‐up of seven surgical patients, six cases were in good condition. Unfortunately, one patient died despite surgery.

In summary, DOLV is a complex and rare anatomical irregularity. With atypical clinical presentations, early and accurate diagnosis can be achieved using TTE, leading to prompt decision‐making in terms of the most suitable surgical interventions when combined with CTA. This, in return, enables anatomical correction and significantly enhances the patient's prognosis.

With all 10 pediatric patients undergoing comprehensive echocardiographic evaluations, and 6 of them successfully undergoing surgical intervention at our center, we generated a comprehensive dataset that encompasses preoperative planning, intraoperative procedures, and postoperative outcomes. This summary provides valuable insights into this clinically rare disease, offering a comprehensive overview of its management and outcomes.

## 5 Conclusion

We present 10 suspected cases of patients with DOLV. The study demonstrates that TTE has a relatively high diagnostic accuracy for DOLV in pediatrics, which is very valuable for its early detection. The combined use of TTE and CTA can enhance the preoperative diagnostic accuracy of DOLV and associated cardiovascular anomalies, respectively. This integrated approach provides a comprehensive assessment that is crucial in formulating clinical management plans and surgical decision‐making processes. Furthermore, TTE is proved to be an invaluable tool for postoperative follow‐up and continued clinical management.

## 6 Limitations

There are some limitations to this study. Firstly, the research data originated from a single medical center. The sample size included in the study is limited because of the rarity of DOLV. Secondly, this was a retrospective study. The clinical data of some cases were incomplete, such as surgical data and postoperative follow‐up data. In future studies, stricter follow‐up should be considered.

## Author Contributions

Xu Zhu and Rui Li conceived and designed the project. Huina Yan and Guangyi Zhong collected and analyzed clinic data of the case. Xu Zhu, Xiaojuan Ji, Xue Xiang, and Min Zheng participated in the diagnosis. Xu Zhu prepared the manuscript, and all authors have read and approved the manuscript as submitted.

## Ethics Statement

This retrospective study was approved by the research ethics committee at Children's Hospital of Chongqing Medical University (2023, 511).

## Consent

Informed consent was waived due to the retrospective design.

## Conflicts of Interest

The authors declare no conflicts of interest.

## Data Availability

All data are presented within the article.
